# Neoadjuvant Chemotherapy Followed by Radical Cytoreductive Surgery in Serous Endometrial Carcinoma With Inguinal Lymph Node Metastasis: A Case Report

**DOI:** 10.7759/cureus.106702

**Published:** 2026-04-09

**Authors:** Yevhenii Maistrenko, Olena Manzhura, Oksana Kravchuk

**Affiliations:** 1 Department of Gynecologic Oncology, Kyiv City Clinical Oncology Center, Kyiv, UKR

**Keywords:** cytoreductive surgery, endometrial cancer, gynecologic oncology, inguinal lymph node metastasis, neoadjuvant chemotherapy, serous endometrial carcinoma, uterine serous carcinoma

## Abstract

Serous endometrial carcinoma is an aggressive histologic subtype of endometrial cancer associated with early dissemination and poor prognosis. Typical metastatic pathways involve pelvic and para-aortic lymph nodes as well as peritoneal surfaces, whereas metastasis to inguinal lymph nodes is extremely uncommon. We report the case of a 42-year-old woman diagnosed with serous endometrial carcinoma confirmed by histopathological and immunohistochemical evaluation of endometrial aspiration biopsy. Imaging revealed metastatic right inguinal lymph nodes measuring up to 21 mm without evidence of additional distant metastases. The patient received four cycles of neoadjuvant chemotherapy consisting of carboplatin and paclitaxel, followed by extensive cytoreductive surgery including radical hysterectomy (type C1), bilateral inguinal lymph node dissection, para-aortic lymphadenectomy up to the level of the left renal vein, and omentectomy. Final histopathological evaluation demonstrated no residual viable tumor, indicating a complete pathological response. At the 12-month follow-up, the patient remains disease-free with no evidence of recurrence. This case highlights the importance of individualized multimodal treatment strategies combining systemic chemotherapy and radical surgery in patients with aggressive histologic subtypes of endometrial carcinoma presenting with atypical metastatic patterns.

## Introduction

Endometrial cancer is the most common gynecologic malignancy in developed countries [1]. Current international guidelines emphasize the importance of accurate staging and individualized treatment strategies in the management of endometrial carcinoma [2].

Serous endometrial carcinoma represents a highly aggressive histologic subtype associated with significantly poorer prognosis compared with endometrioid tumors [3,4]. Because of its aggressive biological behavior, systemic chemotherapy and multimodal treatment approaches are frequently required in advanced disease [5,6].

Lymphatic dissemination commonly involves pelvic and para-aortic lymph nodes, as well as peritoneal surfaces [7]. Contemporary clinical practice guidelines also highlight the importance of comprehensive surgical staging and cytoreductive surgery in selected patients with advanced disease [8].

Metastasis to the inguinal lymph nodes is extremely rare and represents an unusual pattern of lymphatic spread in endometrial carcinoma [9].

## Case presentation

**Figure 1:**
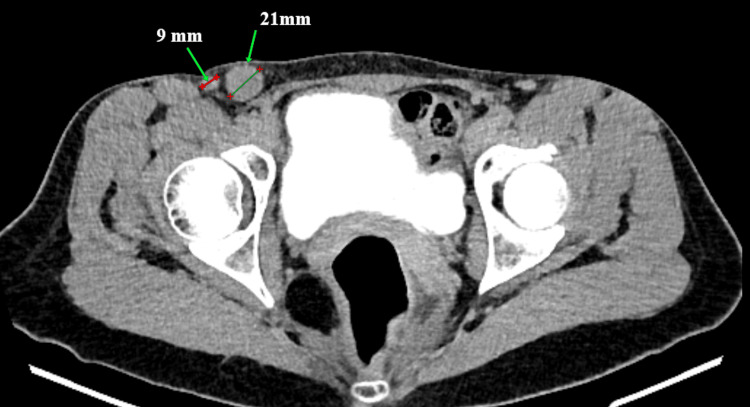
Pre-treatment computed tomography scan demonstrating the enlarged right inguinal lymph nodes Axial computed tomography image demonstrating multiple enlarged and structurally altered right inguinal lymph nodes prior to neoadjuvant chemotherapy. The largest lymph node measured up to 21 mm, consistent with metastatic involvement.

A 42-year-old woman presented with abnormal uterine bleeding. Computed tomography (CT) performed in 10/25 demonstrated multiple enlarged and structurally altered right inguinal lymph nodes measuring 21 mm, 7 mm, 20 mm, and 17 mm, while the left inguinal lymph nodes measured up to 8 mm (Figure 1). Contrast-enhanced CT demonstrated enlarged right inguinal lymph nodes without evidence of additional distant metastases.

**Figure 2:**
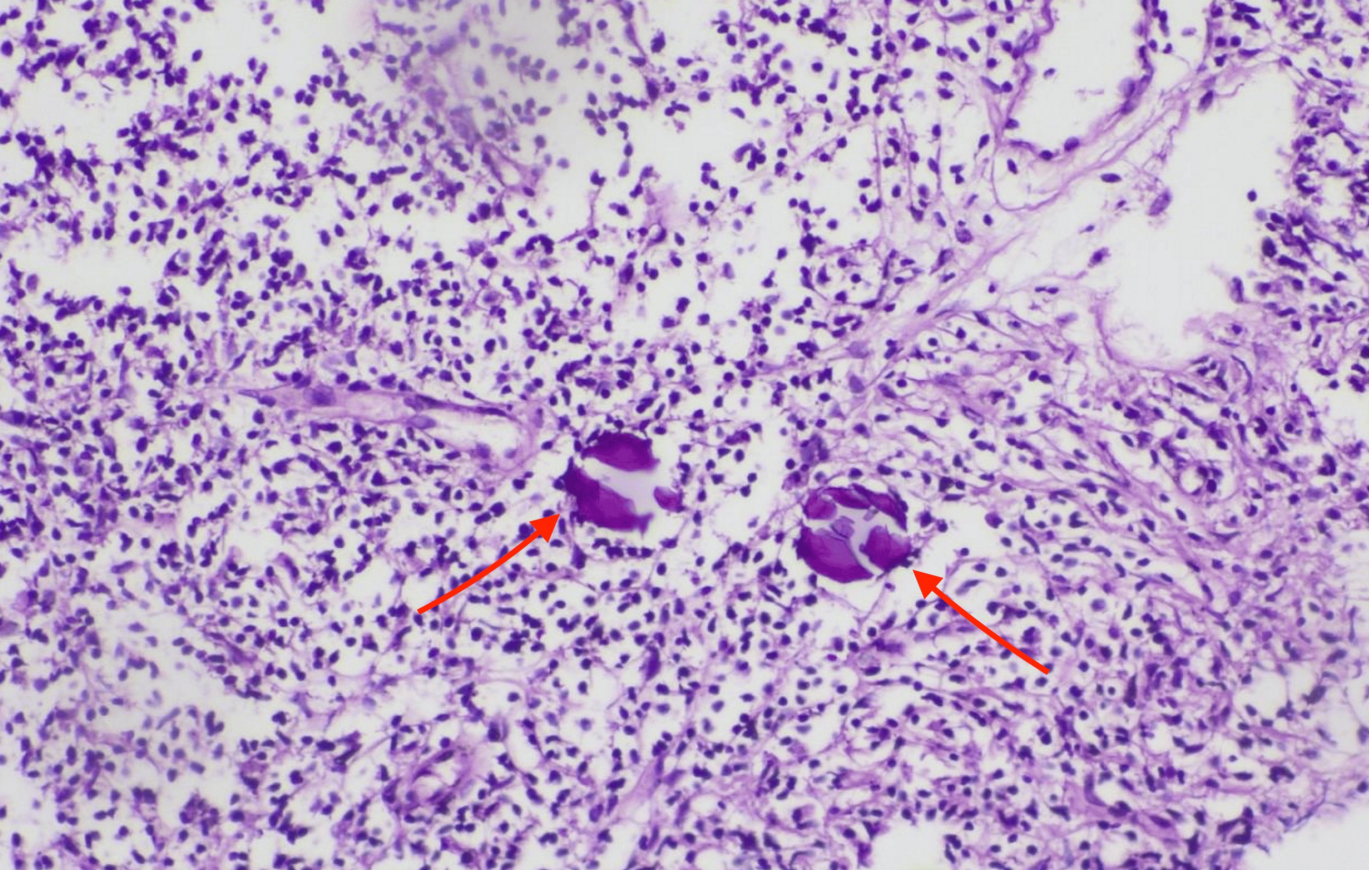
Histopathological examination of inguinal lymph node biopsy Histopathological image of the core needle biopsy specimen from the right inguinal lymph node demonstrating papillary tumor structures composed of atypical epithelial cells consistent with metastatic serous carcinoma. Arrows indicate psammoma bodies. Hematoxylin and eosin stain; total magnification ×40.

**Figure 3:**
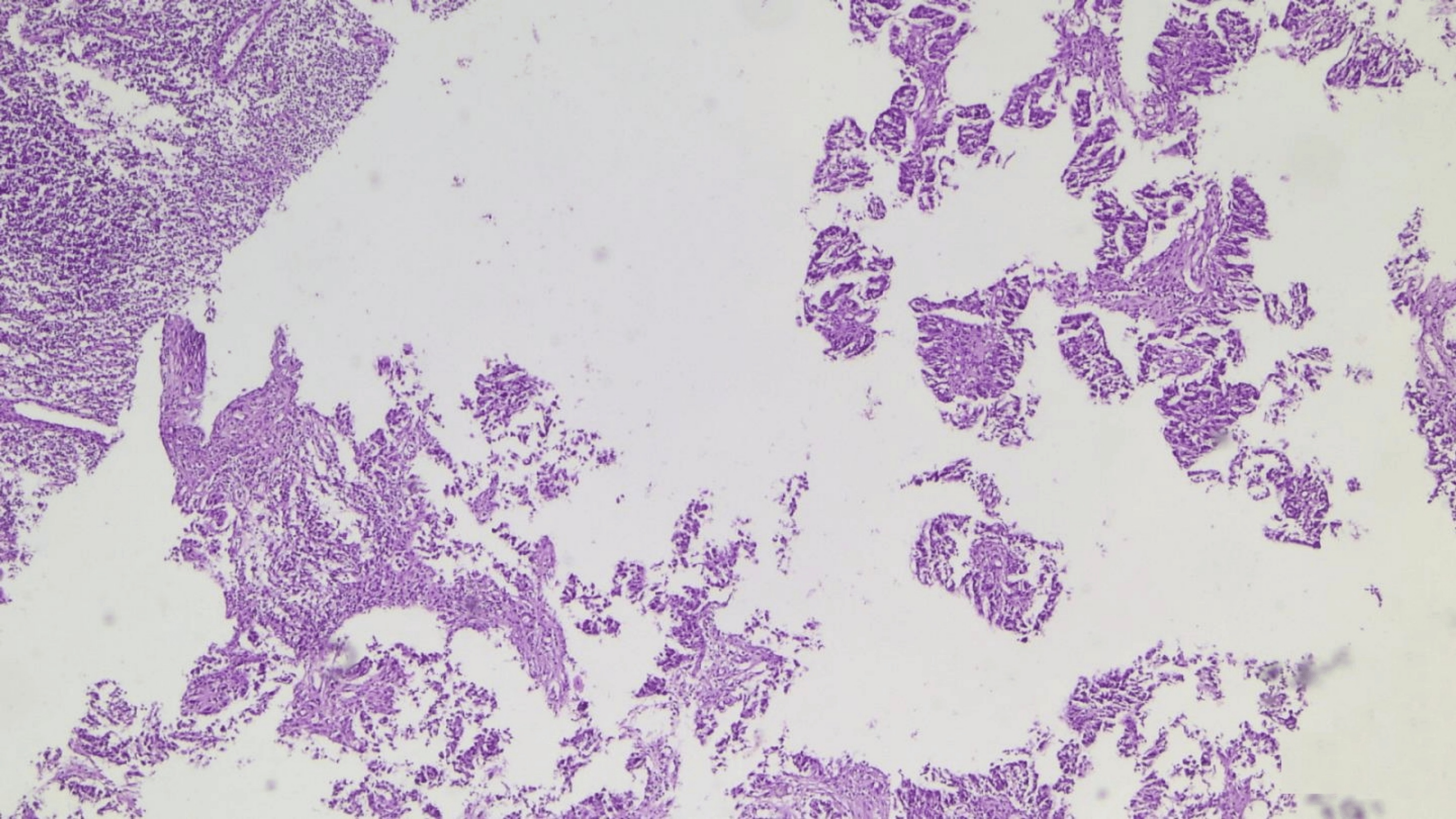
Psammoma bodies in metastatic serous carcinoma Microscopic view demonstrating characteristic psammoma bodies within metastatic serous carcinoma in the inguinal lymph node biopsy specimen. Hematoxylin and eosin stain; total magnification ×40.

In 10/25, a trephine biopsy of the right inguinal lymph node was performed. Histopathological examination confirmed metastatic serous carcinoma. Microscopic evaluation demonstrated papillary tumor structures composed of atypical epithelial cells and the presence of characteristic psammoma bodies (Figures 2-3).

Further evaluation included aspiration biopsy of the endometrium with immunohistochemical analysis performed in 10/25.

The immunohistochemical profile showed negative CD66e expression, positive p16 expression, estrogen receptor expression in 60% of tumor cells, progesterone receptor expression in 5% of tumor cells, positive cytokeratin 7 expression, mutant-type p53 expression, a Ki-67 proliferation index of 60%, and positive WT1 expression. These findings were consistent with serous adenocarcinoma of the endometrium. 

Mismatch repair (MMR) immunohistochemistry was not performed. The diagnosis was established based on characteristic morphology and immunohistochemical profile, including mutant-type p53 expression, and additional molecular testing was not considered to impact clinical management in this case.

Differential diagnoses, including ovarian serous carcinoma and other Müllerian primary tumors, were considered. The absence of an ovarian mass on imaging, together with the immunohistochemical profile and concordant endometrial biopsy findings, supported a primary endometrial origin of the tumor.

Based on imaging findings and histopathological confirmation of nodal involvement, the disease was interpreted as metastatic endometrial carcinoma (International Federation of Gynaecology and Obstetrics (FIGO) stage IVB), which informed treatment planning and the decision to initiate neoadjuvant chemotherapy.

Following a multidisciplinary tumor board discussion involving specialists in gynecologic oncology, radiology, pathology, and medical oncology, the case was reviewed in detail. The discussion focused on the extent of disease, the presence of atypical inguinal lymph node metastasis, and the feasibility of achieving optimal cytoreduction. Based on this evaluation, a treatment strategy consisting of neoadjuvant chemotherapy followed by radical cytoreductive surgery was selected.

Positron emission tomography (PET)/CT was not performed in this case. Contrast-enhanced CT was used as the primary imaging modality, as it was considered sufficient to assess disease extent and guide treatment planning.

**Figure 4:**
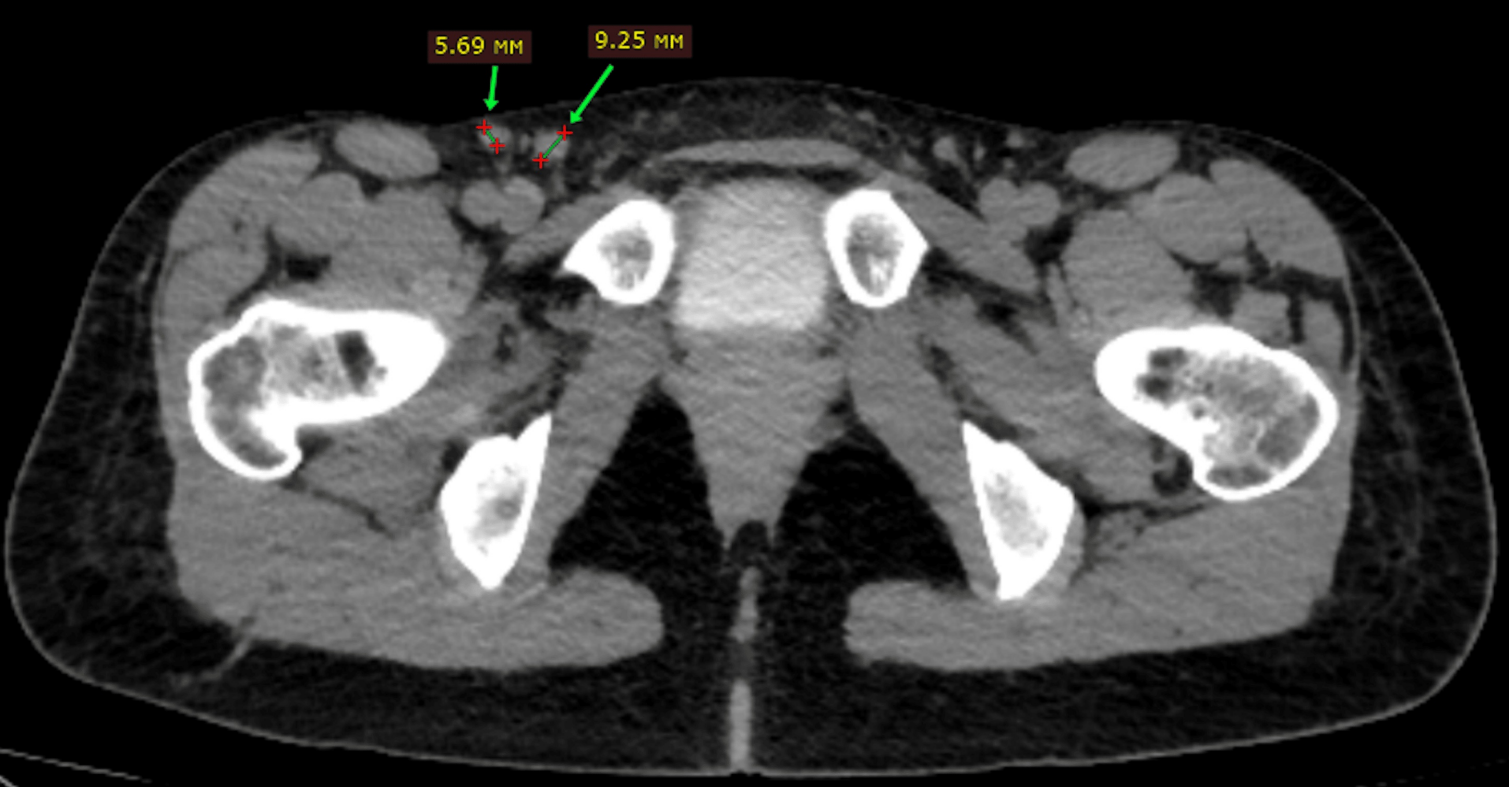
Computed tomography after neoadjuvant chemotherapy demonstrating the regression of the inguinal lymph nodes Axial computed tomography image obtained after four cycles of neoadjuvant chemotherapy with carboplatin and paclitaxel demonstrating the regression of previously enlarged right inguinal lymph nodes.

The patient received four cycles of neoadjuvant chemotherapy consisting of carboplatin and paclitaxel. Repeat imaging demonstrated regression of the previously enlarged inguinal lymph nodes (Figure 4).

Subsequently, extensive cytoreductive surgery was performed, including radical hysterectomy (type C1), bilateral inguinal lymph node dissection, para-aortic lymphadenectomy up to the level of the left renal vein (PALND2), and omentectomy. 

The decision to perform a type C1 radical hysterectomy despite the presence of inguinal lymph node metastasis was based on the favorable response to neoadjuvant chemotherapy and the feasibility of achieving complete cytoreduction. The surgical approach aimed to achieve no residual disease (R0 resection), which is a key prognostic factor in advanced endometrial carcinoma.

**Figure 5:**
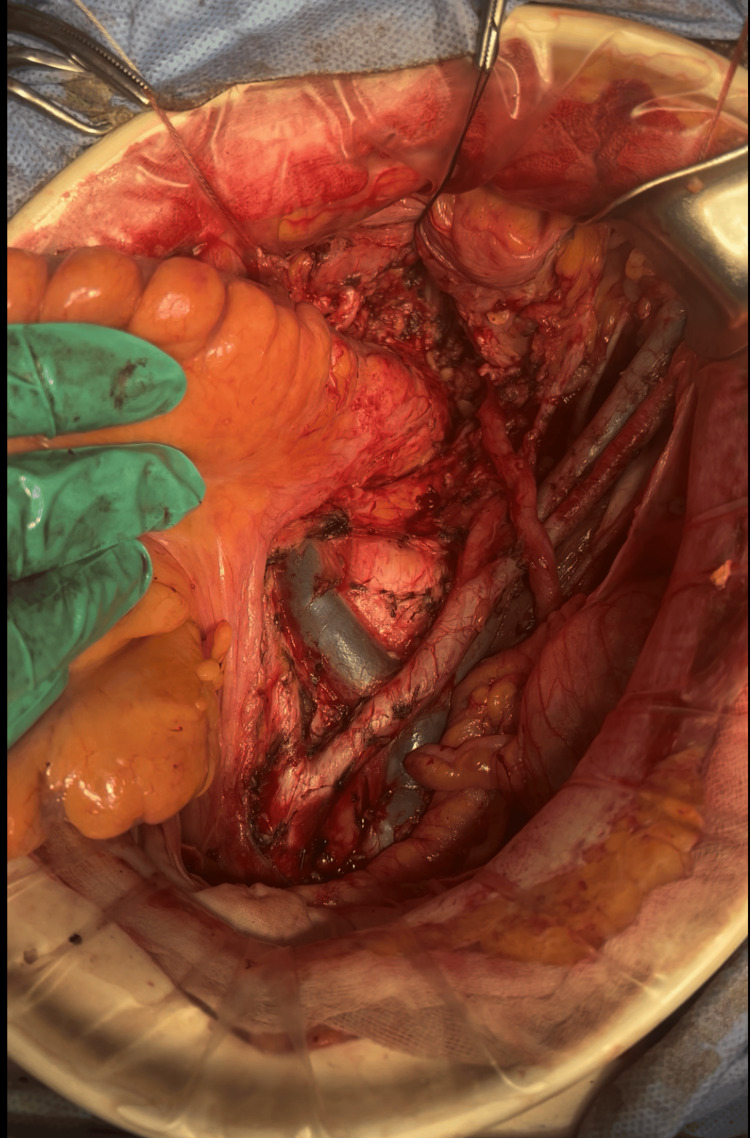
Intraoperative view during para-aortic lymphadenectomy Intraoperative image demonstrating para-aortic lymph node dissection up to the level of the left renal vein. The abdominal aorta and inferior vena cava are exposed following the removal of surrounding lymphatic and adipose tissue during radical cytoreductive surgery.

Para-aortic lymph node dissection was performed with exposure of the inferior vena cava and abdominal aorta following the removal of surrounding lymphatic and adipose tissue (Figure 5).

**Figure 6:**
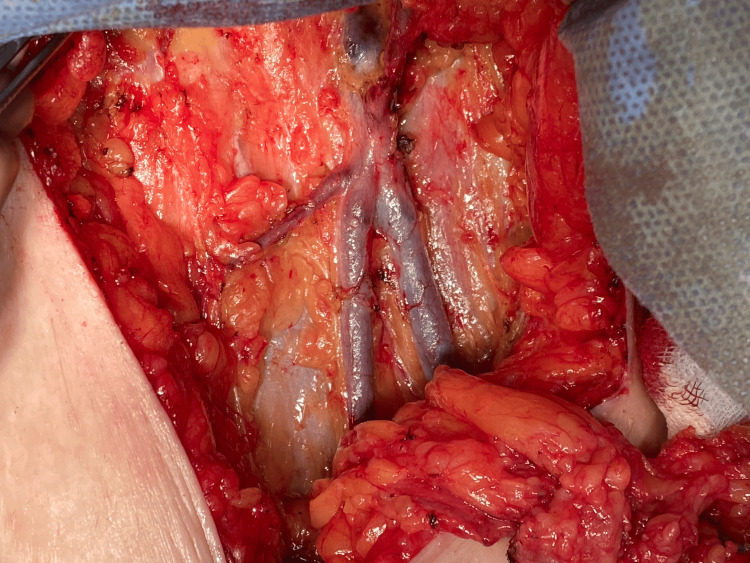
Intraoperative view of right inguinal lymph node dissection Intraoperative image demonstrating right inguinal lymph node dissection with exposure of the femoral vessels during cytoreductive surgery for serous endometrial carcinoma.

Right inguinal lymph node dissection was then performed with careful exposure of the femoral vessels (Figure 6).

**Figure 7:**
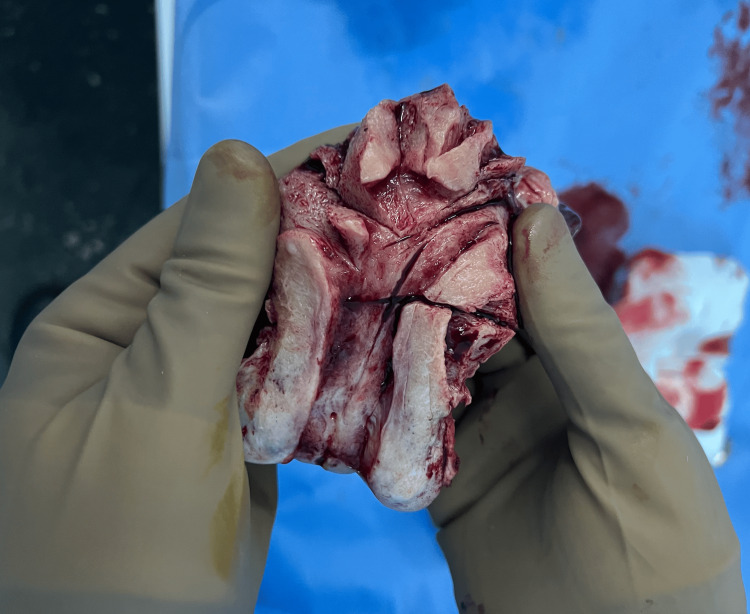
Gross cross-section of the uterus after neoadjuvant chemotherapy Macroscopic examination of the uterine specimen following radical hysterectomy demonstrating no visible residual tumor within the endometrial cavity after neoadjuvant chemotherapy.

Macroscopic examination of the uterine specimen demonstrated no visible tumor within the endometrial cavity (Figure 7).

**Figure 8:**
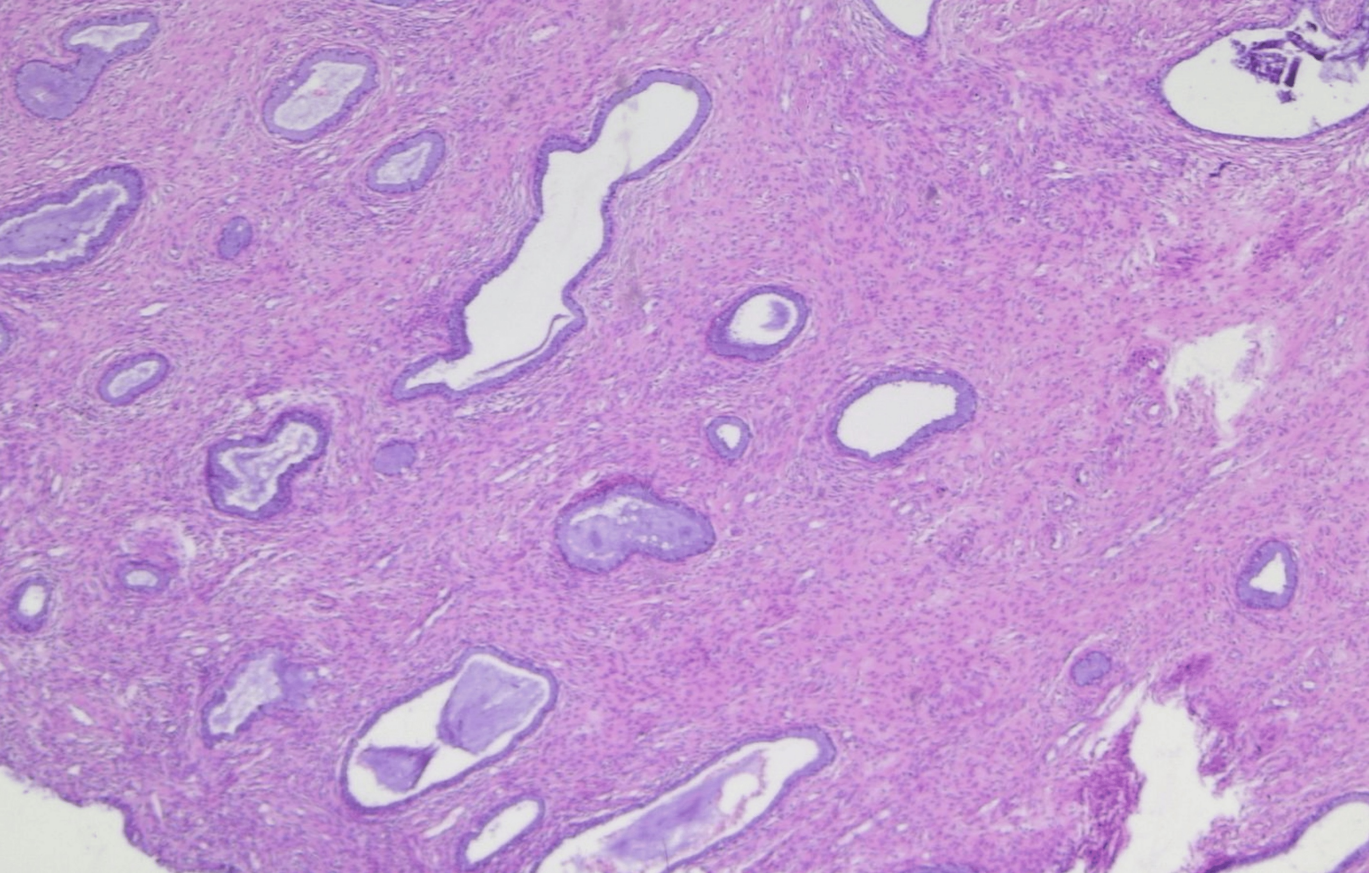
Endometrium after neoadjuvant chemotherapy Microscopic image demonstrating proliferative-type endometrium without cytological atypia or residual carcinoma, confirming complete pathological response following neoadjuvant chemotherapy. Hematoxylin and eosin stain, total magnification ×40.

The postoperative course was uneventful. Final histopathological examination demonstrated no residual viable tumor. The endometrium showed proliferative-type changes without evidence of malignancy (Figure 8).

**Figure 9:**
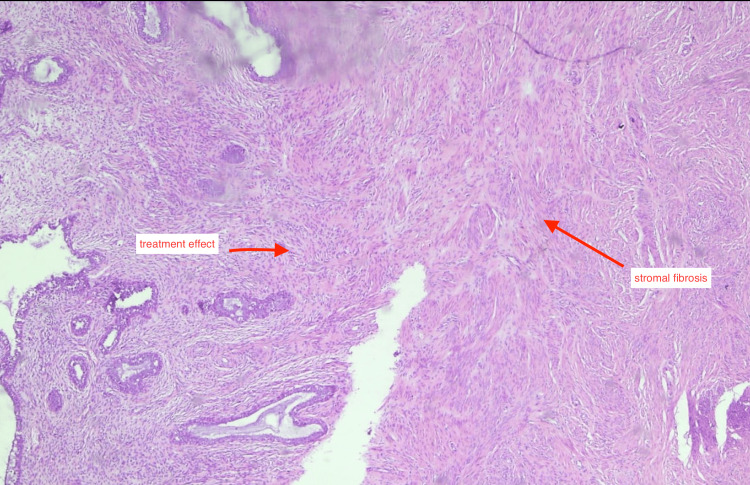
Treatment-related changes in the endometrium Histopathological image showing marked stromal fibrosis and treatment-related changes without evidence of viable tumor cells, supporting treatment effect. Hematoxylin and eosin stain, total magnification ×100.

The ovaries demonstrated fibrous bodies, and the omentum showed vascular congestion. Pelvic, para-aortic, aortocaval, and left inguinal lymph nodes revealed sinus histiocytosis, lipomatosis, and fibrosis without evidence of tumor involvement. Right inguinal lymph nodes demonstrated deposition of psammoma bodies without viable tumor cells, consistent with treated metastatic disease. These findings were consistent with a complete pathological response to neoadjuvant chemotherapy (Figure 9).

At the 12-month follow-up, the patient remains disease-free with no clinical or radiological evidence of recurrence (Table 1).

**Table 1:** Chronological timeline of diagnostic and therapeutic interventions CT: computed tomography; IHC: immunohistochemistry; LND: lymph node dissection; PALND2: para-aortic lymphadenectomy up to the level of the left renal vein

Time point	Event	Details
Day 0	Presentation	Abnormal uterine bleeding
Day 3	Imaging (CT)	Enlarged right inguinal lymph nodes without other distant metastases
Day 5	Inguinal lymph node biopsy	Metastatic serous carcinoma
Day 7	Endometrial biopsy + IHC	Serous endometrial carcinoma (p53 mutant, WT1+, ER/PR+)
Day 14	Treatment initiation	Neoadjuvant chemotherapy (carboplatin + paclitaxel)
Days 14-90	Systemic therapy	4 cycles neoadjuvant chemotherapy
Day 90	Surgery	Radical hysterectomy (C1), bilateral inguinal LND, PALND2, omentectomy
Postoperative	Histopathology	Complete pathological response
Adjuvant phase	Additional treatment	2 cycles adjuvant chemotherapy + radiotherapy
Follow-up	Outcome	No evidence of disease at 12 months

## Discussion

Serous endometrial carcinoma is an aggressive subtype associated with a high risk of lymphovascular invasion and distant metastasis [4]. Inguinal lymph node involvement represents an unusual pattern of dissemination and is reported in less than 1% of cases, corresponding to FIGO stage IVB disease.

In this case, the presence of an isolated inguinal lymph node metastasis required careful diagnostic evaluation and multidisciplinary decision-making. The combination of imaging findings and histopathological confirmation established advanced-stage disease, guiding the selection of systemic therapy.

Neoadjuvant chemotherapy with carboplatin and paclitaxel was chosen to reduce tumor burden and improve the feasibility of complete cytoreduction. Following a favorable response, extensive surgery was performed with the aim of achieving no residual disease (R0 resection), which is a key prognostic factor in advanced endometrial carcinoma [2,5,8].

A notable finding in this case was the complete pathological response, with no viable tumor cells identified in the uterus or lymph nodes. The presence of psammoma bodies in previously involved inguinal lymph nodes likely reflects treatment-related changes and residual evidence of regressed serous carcinoma.

From a clinical perspective, this case highlights several important considerations. First, atypical sites of lymphatic spread, such as inguinal lymph nodes, should be recognized as possible manifestations of advanced disease. Second, neoadjuvant chemotherapy may be an effective strategy in selected patients with unusual metastatic patterns, enabling optimal cytoreductive surgery. Finally, a multidisciplinary approach is essential for individualized treatment planning in rare and complex presentations.

Further accumulation of similar cases is needed to better define optimal management strategies for this rare pattern of metastatic spread.

## Conclusions

Serous endometrial carcinoma with inguinal lymph node metastasis represents a rare clinical presentation corresponding to FIGO stage IVB disease. This case demonstrates that a multimodal treatment strategy combining neoadjuvant chemotherapy and radical cytoreductive surgery can achieve complete pathological response and favorable short-term oncologic outcomes.

Careful evaluation of atypical lymph node involvement and individualized, multidisciplinary treatment planning are essential in managing aggressive histologic subtypes of endometrial carcinoma.

The key clinical takeaway is that atypical sites of lymphatic spread, such as inguinal lymph nodes, should not preclude aggressive multimodal management, as favorable outcomes may still be achieved with appropriate patient selection. 
